# Personalized additive manufacturing of devices for the management of enteroatmospheric fistulas

**DOI:** 10.1002/btm2.10583

**Published:** 2023-09-26

**Authors:** Francisco José Calero Castro, Andrés Padillo Eguía, Virginia Durán Muñoz‐Cruzado, Luis Tallón Aguilar, José Tinoco González, Imán Laga, Fernando de la Portilla de Juan, Felipe Pareja Ciuró, Javier Padillo Ruiz

**Affiliations:** ^1^ Oncology Surgery, Cell Therapy, and Organ Transplantation Group, Instituto de Biomedicina de Sevilla (IBiS) Hospital Universitario Virgen del Rocío, CSIC, Universidad de Sevilla Seville Spain; ^2^ Department of General Surgery IBiS, Hospital University Virgen del Rocío, CSIC, University of Seville Seville Spain; ^3^ University of Seville Seville Spain

**Keywords:** 3D printing, computer‐aided design, digestive system fistula, personalized medicine, wound care, wound healing

## Abstract

Additive manufacturing techniques allow the customized design of medical devices according to the patient's requirements. Enteroatmospheric fistula is a pathology that benefits from this personalization due to its extensive clinical variability since the size and morphology of the wound differ extensively among patients. Standard prosthetics do not achieve proper isolation of the wound, leading to a higher risk of infections. Currently, no effective personalized technique to isolate it has been described. In this work, we present the workflow for the design and manufacture of customized devices adapted to the fistula characteristics as it evolves and changes during the treatment with Negative Pressure Wound Therapy (NPWT). For each case, a device was designed with dimensions and morphology depending on each patient's requirements using white light scanning, CAD design, and additive manufacturing. The design and manufacture of the devices were performed in 230.50 min (184.00–304.75). After the placement of the device, the wound was successfully isolated from the intestinal content for 48–72 h. The therapy was applied for 27.71 ± 13.74 days, and the device was redesigned to adapt to the wound when geometrical evolutionary changes occur during the therapy. It was observed a decrease in weekly cures from 23.63 ± 10.54 to 2.69 ± 0.65 (*p* = 0.001). The fistulose size was reduced longitudinal and transversally by 3.25 ± 2.56 cm and 6.06 ± 3.14 cm, respectively. The wound depth also decreased by 1.94 ± 1.08 cm. In conclusion, customization through additive manufacturing is feasible and offers promising results in the generation of personalized devices for the treatment of enteroatmospheric fistula.

**Translational Impact Statement**

Enteroatmospheric fistula is a complex pathology that has an extensive clinical variability, where a gastrointestinal tract is contact with an open abdominal wound. Part of its treatment is based on Negative Pressure Wound Therapy and the wound has to be isolated from the intestinal debit. Thereby, it requires a personalized device that is designed with measurement taken by a scan from the each patient wound. The relevance of this invention is that we could personalized the treatment of each patient with a different device which reduce the weekly cures and the wound size.

## 1 INTRODUCTION

Enteroatmospheric fistula is a complex pathology that usually results from a post‐surgical complication. Anatomically, it consists of a communication between the gastrointestinal tract and the surface of an open abdominal wound.^1^, ^2^, ^3^ Although its main treatment is very complex, one of the fundamental aspects is the local management of the wound. Its initial treatment primarily depends on local control of the wound. Therefore, it is necessary to have a device that, associated with Negative Pressure Wound Therapy (NPWT), prevents the intestinal contents to be in contact with the wound, to avoid contamination and promote healing. Wound management is very complex due to the extensive clinical variability they present, involving different intestinal tracts and a variable number of fistulous orifices on the surface of the wound, through which the intestinal debit from the abdominal cavity is collected.^2^ Another characteristic of this pathology is its variability over time, which means that a patient requires different devices during the healing process, either because the abdominal wound becomes smaller or because the intestinal surface becomes larger during the healing process. So far, different techniques have been described that aim to isolate the intestinal contents of the wound from the abdominal wall, so that the wound is not in contact with the intestinal fluid and the wound can be healed. Different techniques allow the isolation and the correct granulation of the wound, as they help to create a “floating stoma.”^2^ However, these devices have a standard size and they are not valid for all types of fistulas, since as we mentioned before, the wounds present a high clinical variability and different sizes. Thus, personalized treatment is required.

Additive manufacturing is one of the most revolutionary and powerful techniques from the last decades. It is known for creating prototypes and structures quickly and economically with a fine degree of resolution.^4^ The 3D printing industry has allowed great advances in medical equipment, implant material, and cell printing,^5^ paving the way for the rapid printing of artificial devices or prostheses by facilitating tissue regeneration. It allows personalized treatments to be achieved, due to the additive manufacturing of biomaterials and bio‐printing that facilitate the creation of customized devices designed by assisted design.^4^

Several techniques for image acquisition aimed toward their application for personalized medicine have been described, such as computerized tomography (CT) or magnetic resonance image (MRI), and have proven to be accurate for this task. However, they present some important drawbacks, such as the radiation exposure of the patient, in the case of CT, or the time‐consuming process that is image acquisition by MRI. Therefore, the analysis of the continuously changing wound morphology and the replacement of the device for proper wound isolation and treatment is not feasible with these image acquisition techniques.^6^ In our study, we present a white light scanner as an accurate, fast, and less invasive technique for wound image acquisition than the ones previously mentioned.

Techniques based on structured‐light scanners and inverse engineering allow the 3D reconstruction of the abdominal area and provide the necessary parameters for the personalization of the devices for the fistula treatment, without the inconveniences presented by other mentioned techniques.

To date, no work has been found in the available literature describing the use of a white light scanner for image acquisition specifically targeted toward personalized medical devices. Despite their use in medicine for measuring limb volumes,^7^ they have not been used yet to define the surface of a complex wound. Their application on enteroatmospheric fistula treatment is especially relevant since they allow a high level of personalization based on the patient's anatomy.

We propose a standardized methodology for the personalization of adapters obtained through additive manufacture for the treatment of enteroatmospheric fistulas according to each patient's needs.

## 2 RESULTS

### 2.1 Surface reconstruction

**FIGURE 1:**
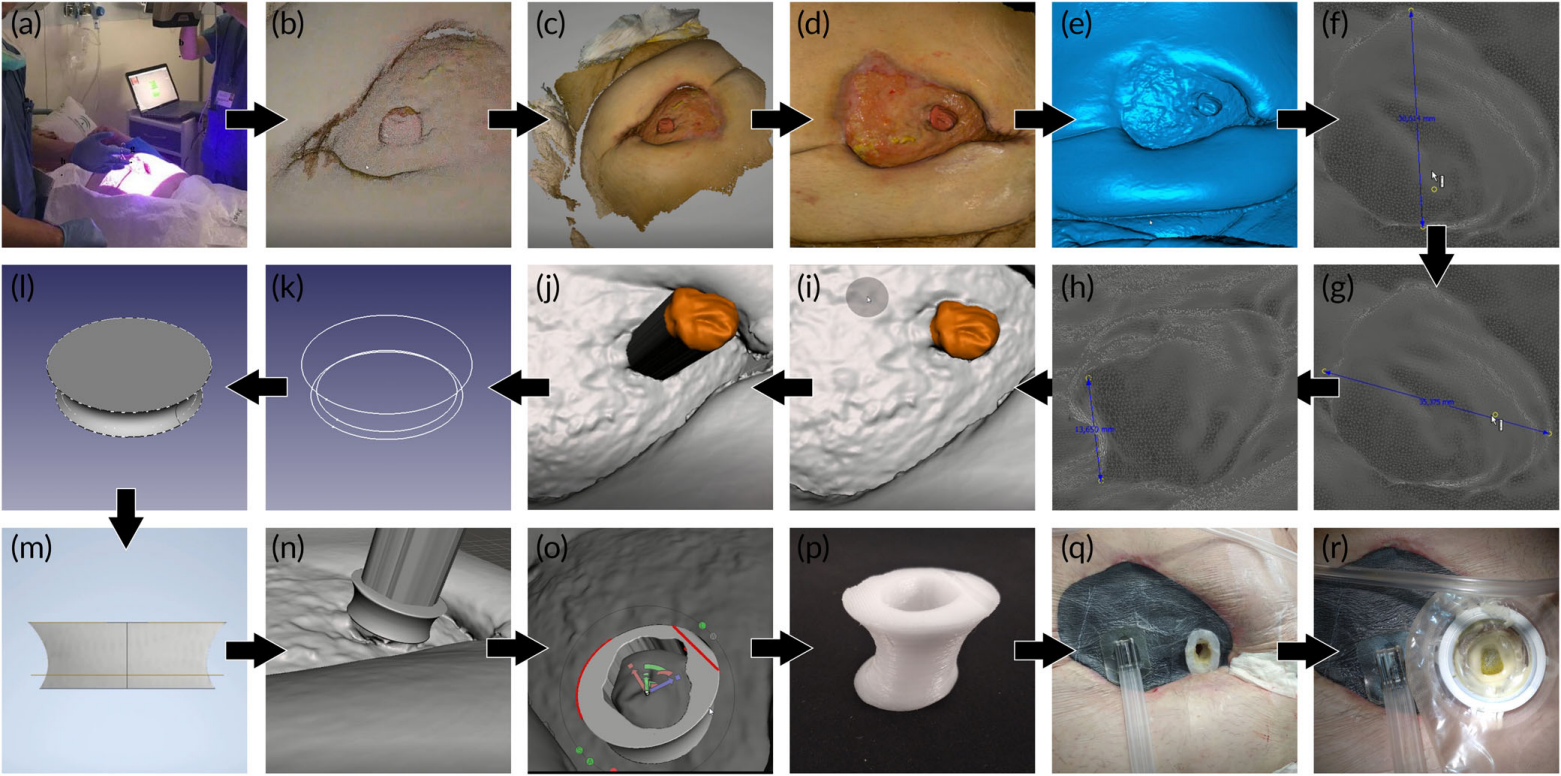
Fabrication devices process. **(**a). Scanning process. (b,c). Point cloud. (c) Point cloud. (d,e). Wound reconstruction. (f–h). Fistula measurement process. (i) Fistula selection. (j) Fistula extrusion. (k) Sketches in different planes. (l–m) Device design. (n) Boolean subtraction. (o) Device design. (p) Fabrication device. (q) Placement device with NPWT therapy. (r) Floating ostomy.

The measurements of two patients were taken manually. The rest of the patients were scanned at least once. Six patients were scanned and virtually reconstructed for the design of the customized devices. Two patients of the six were measured manually twice before taking the measurement digitally and the rest of the scanned patients (four of the six mentioned patients) required a re‐scanning. 288,271.00 ± 96,765.24 points were captured, and 222,111.20 ± 59,604.00 were processed. Finally, the generated meshes had 703,991.80 ± 217,443.82 points and 1,408,222.40 ± 434,944.74 triangles. Figure 1 shows the process from the scanning to the placement of the devices. Manual measurement took 4.08 ± 2.29 min, whereas digital measurement lasted 21.00 ± 6.99 min, with significant differences between both (*p* < 0.001).

### 2.2 Device design

The design of the six devices with manual measurement took a mean time of 33.33 ± 26.96 min. The mean time value for the 10 designs with virtual reconstruction was 37.00 ± 17.67 min. There were no significant differences in the device design time between manual measurements and virtual reconstruction (*p* = 0.746).

### 2.3 Additive manufacturing of the device

Table 1 shows the characteristic of each device. The mean height of the devices was 1.48 ± 0.36 cm, the width was 2.47 ± 1.49 cm, and the length was 3.37 ± 2.29 cm. The upper protrusion has a mean size of 0.82 ± 0.33 cm, while the lower protrusion was 0.57 ± 0.31 cm. The time spent in the 3D printing, post‐processing, and sterilization were 124.50 min (56.75, 167.25) and 63.13 ± 12.92 min, respectively.

**TABLE 1:** Device characteristics.

Device	Taking measurement	Point cloud	2° Point cloud	Number of final points	Number of triangles	Height	Width	Length	Top ledge	Bottom ledge	Measure time	Design time	Manufacturing time	Post‐processing time	Total time	
1	Manual	–	–	–	–	2	6	9	1.5	1	4.5	60	390	70	524.5	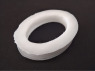
2	Manual	–	–	–	–	2	3	8	1.5	1	4	65	325	65	459	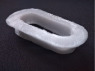
3	Manual	–	–	–	–	1	1	1	0.3	0.3	6	20	45	60	131	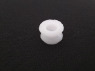
4	Manual	–	–	–	–	1	1.5	1.5	1	1	7	5	40	60	112	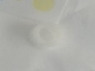
5	Scanner	162,013	132,639	427,434	854,864	1	1.3	2.2	1	1	20	60	38	60	178	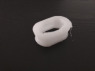
6	Scanner	196,347	188,527	492,409	985,218	1.5	0.8	0.8	0.3	0.2	35	60	18	70	183	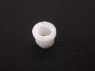
7	Scanner	221,505	219,614	506,203	1,012,526	1.5	3.5	4.5	0.7	0.5	30	50	172	65	317	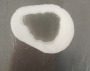
8	Manual	–	–	–	–	2	2	3	0.7	0.3	2	45	115	65	227	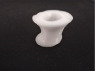
9	Manual	–	–	–	–	2.8	3	3	0.6–0.8	0.2–0.6	1	5	130	65	201	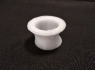
10	Scanner	481,104	349,133	924,731	1,850,126	1.5	3	3.5	0.7	0.3	25	25	153	65	268	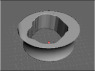
11	Scanner	355,238	254,712	930,626	1,861,428	1.5	2	3	0.4–1	0.2–0.7	20	30	119	65	234	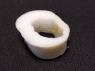
12	Scanner	355,238	254,712	930,626	1,861,428	1.5	1.5	1.5	1	0.5	15	15	110	60	200	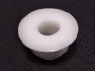
13	Scanner	227,215	163,856	431,593	863,462	1.5	3.5	4.8	1	1	20	55	130	60	265	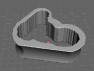
14	Scanner	234,584	191,531	707,240	1,414,580	1.5	2.5	4.8	0.5	0.5	15	35	142	60	252	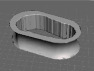
15	Scanner	324,733	233,194	844,528	1,689,296	2	4	5.2	1	0.5	15	20	240	60	335	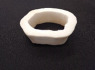
16	Scanner	324,733	233,194	844,528	1,689,296	1.2	1.5	2	1	0.3	15	20	92	60	187	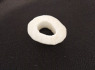

### 2.4 Treatment effectiveness

The personalization of these devices was feasible since it was possible to generate an adapter for each patient (or more adapters in the case of major evolutionary changes) in 230.50 min (184.00–304.75). Regarding the applicability of the device, it was used in all eight patients, fulfilling its function properly. The therapy was applied for 27.71 ± 13.74 days. The time of application of the therapy depended on the evolution presented by the patients and on the need to apply other concomitant therapies to solve this complex pathology. The application of this device has reduced significantly the weekly number of cures in the analyzed patients from 23.63 ± 10.54 to 2.69 ± 0.65 (*p* = 0.001).

**FIGURE 2:**
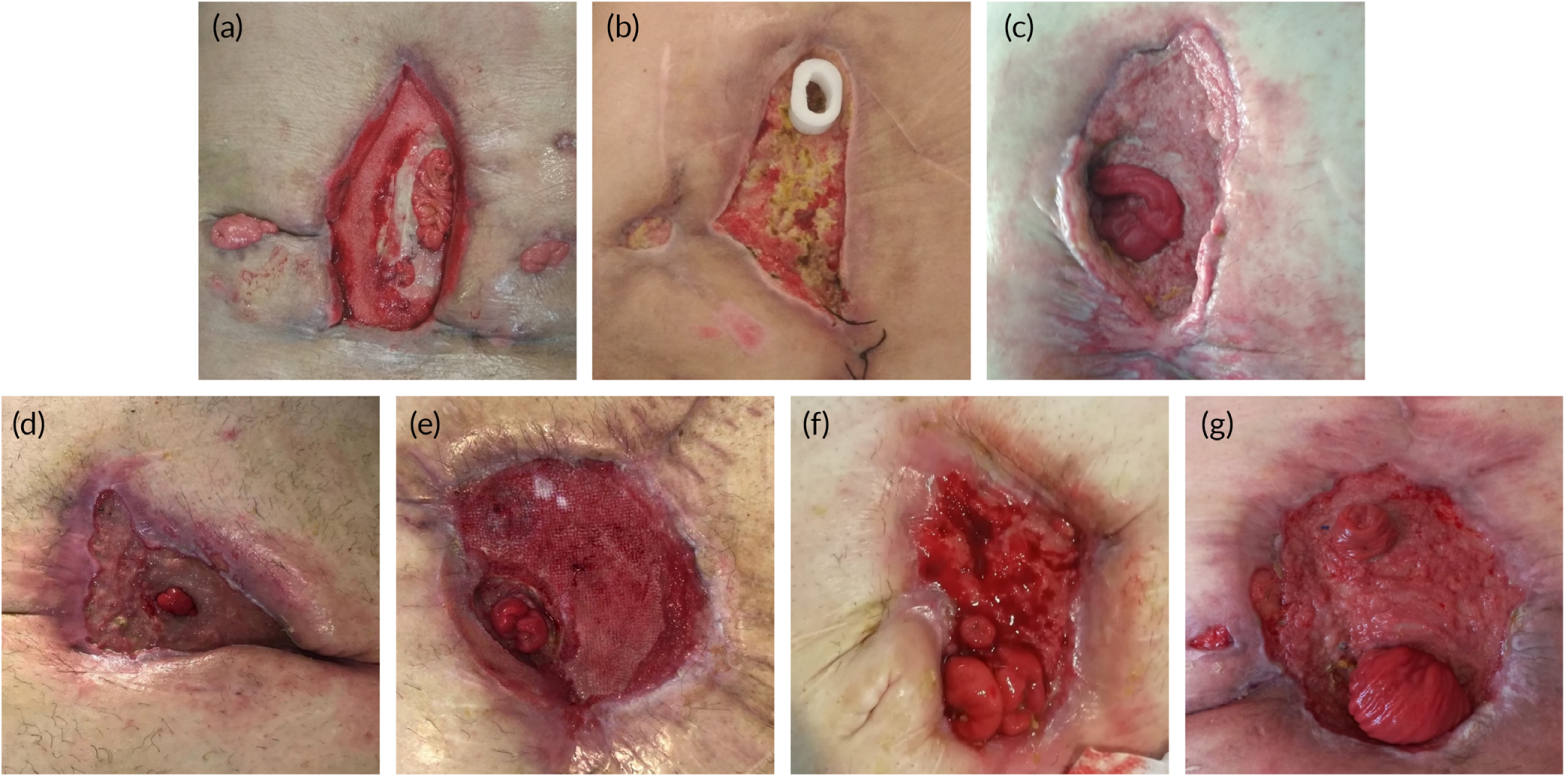
Fistula after therapy. (a) Patient 2; (b) Patient 3; (c) Patient 4; (d) Patient 5; (e) Patient 6; (f) Patient 7; and (g) Patient 8.

The system was kept watertight for an average of 24 h with no need for changes and cures, sometimes remaining 48–72 h with no need for new cures. Thanks to the application of the device associated with the NPWT system, the size of the wound was progressively reduced (Figure 2), since the presence of the adapted device allows the concentration of the pressure on the wound areas that require treatment, without affecting the surrounding surface. Table 2 shows the wound measurements before and after therapy. The average longitudinal measurement was reduced after therapy from 14.00 ± 4.04 to 10.75 ± 4.23 cm (*p* = 0.009), the average transverse measurement from 13.63 ± 4.47 to 7.56 ± 3.06 cm (*p* = 0.001), and the average depth from 2.56 ± 1.45 to 0.63 ± 0.74 cm (*p* = 0.011).

**TABLE 2:** Fistula morphology and clinical symptoms for each patient.

Patient	Length before	Length after	Width before	Width after	Height before	Height after	Cures before (weeks)	Cures after (weeks)	Pruritus before	Pruritus after	VAS before	VAS after	Therapy time (days)	Number of changes	Solution	Surgery for definitive closure	Survival 3 months after admission
1	12	10	14	11	2	1	28	3	6	0	8	4	14	4	Ostomization	No	No
2	16	16	13	8	6	2	28	3.5	7	0	6	0	8	3	Closure	Yes	Yes
3	20	18	4	3	2	1	35	2.5	5	2	7	3	15		Closure	No	No
4	9	7.5	15	4	2	1	35	3.5	6	0	5	0	30	10	Ostomization	Yes	Yes
5	16	9	15	7	2	0	14	2	5	0	0	0	29	8	Ostomization	Yes	Yes
6	16	9	15	8	1.5	0	14	3	0	0	2	0	28	8	Ostomization	Pending	Yes
7	8	5.5	13	7.5	2	0	28	2	5	0	8	0	50	23	Ostomization	Yes	Yes
8	15	11	20	12	3	0	7	2	6	0	7	0	35	13	Ostomization	Pending	Yes

Abbreviation: VAS: visual analogic scale for pain.

The pruritus doses before therapy were 5.50 (5.00–6.00) and the visual analogic scale for pain (VAS) was 5.38 ± 2.92. After the application of the therapy, the pruritus dose was 0 and VAS was 0 (0–2.25). Significant differences in pruritus (*p* = 0.017) and VAS (*p* = 0.018) were demonstrated. Finally, survival at 3 months was 75.00%, and the outcome was an ostomization in 75.00% of the patients, whereas among the rest was the closure surgery (Table 2).

## 3 DISCUSSION

The use of additive manufacturing in medicine can be applied in various fields, ranging from surgical planning in traumatology or urology^8^, ^9^, ^10^ to the creation of orthoses or splints^11^ or even to tissue engineering and regenerative medicine.^12^ Personalized treatment can be easily achieved due to additive manufacturing that allows the creation of devices designed by assisted design.^4^ Currently, our group has achieved this personalization in the fistula treatment by using for the first time a bioscanner to define the wound area on a patient's body.

As a result of the cases studied in this work, we can see the different clinical situations that can arise in the case of an enteroatmospheric fistula and understand the need for the personalization of devices for the treatment of this pathology. The first clinical results of the application of this therapy have been widely described by Durán Muñoz‐Cruzado et al.^13^ from our working group, which corroborates the efficacy and safety of the application of the device generated by the methodology presented in this work.

Some papers^14^, ^15^, ^16^ present images of enterocutaneous fistulas taken from CT. However, when combining CT slices to form a volumetric body, the resolution of the wound surface is lost. Another benefit of the white light scanner is that the image‐obtaining process is harmless to the patients with a higher resolution, in opposition to the CT technique, since no ionizing radiation is employed. Therefore, it can be repeated as much as needed. Additionally, once the bioscanner is bought, there are no additional costs of maintenance. These characteristics make this technique potentially useful not only for the treatment of enteroatmospheric fistulas but also for the design of other medical devices for other pathologies.

Another important advantage of the scanner is the fact that the image acquisition process can take place in the patient's room, avoiding the typical discomfort associated with CT and MRI techniques (claustrophobic feelings, acoustic noise …). Moreover, the efflux cannot be removed in situ from the wound when the images are taken from CT or MRI, whereas it can be cleaned when the patient is being scanned in his room. This is an advantage of the white light scanner that allows us to capture more clearly the fistulous orifices.

**FIGURE 3:**
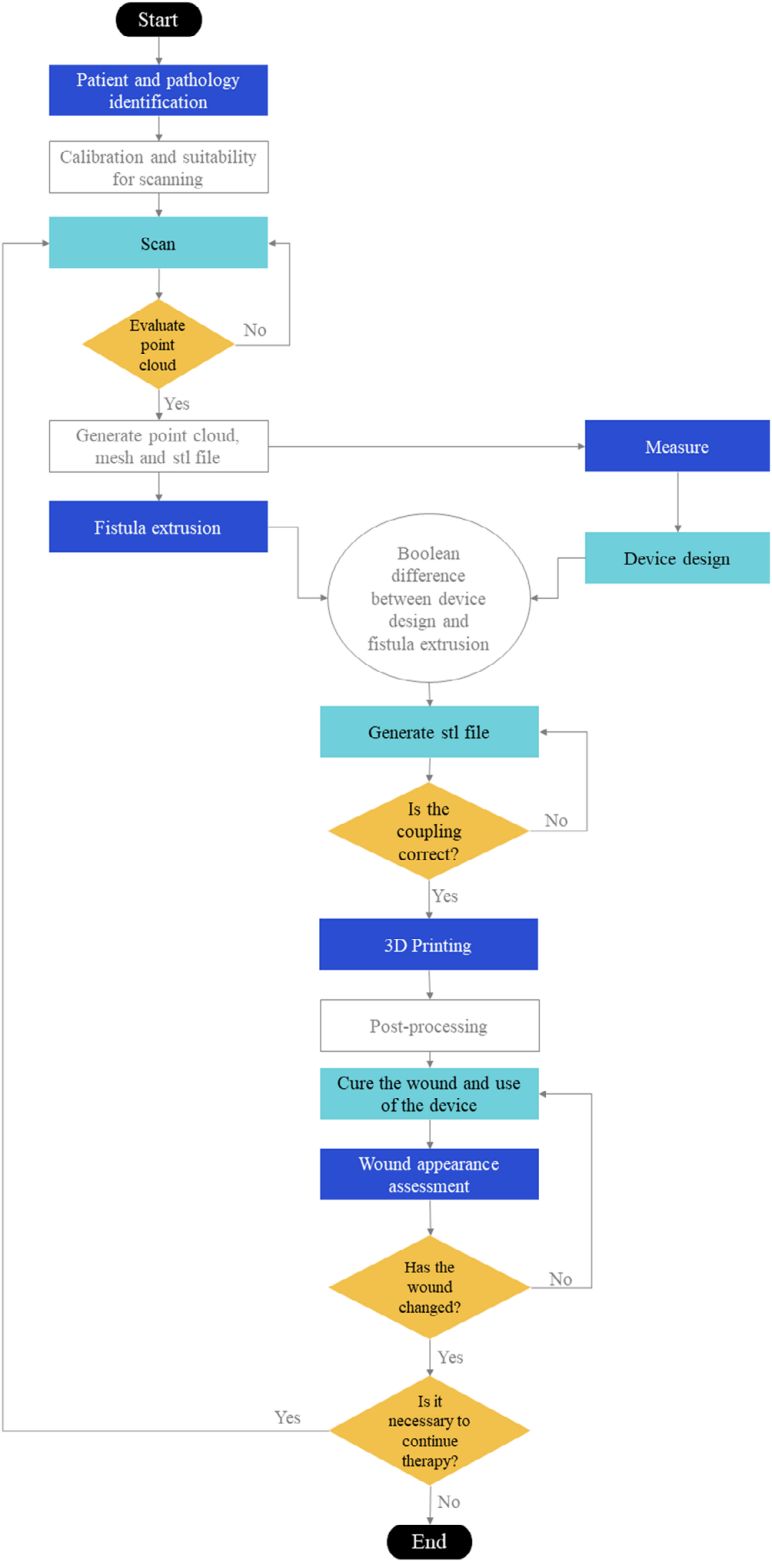
Algorithm.

Not only one image is used to perform the measurements. As indicated in the algorithm in Figure 3, depending on the clinical needs assessed by the surgeon himself, a second and even a third scan of the patient must be performed. These are the reasons why we chose this low‐cost technology that allows the acquisition of as many high‐resolution medical images as necessary with minimal impact on the patient's comfort and safety.

Similar techniques and devices aim to perform the same function as the device we have presented in this work. In 2002, Subramaniam et al.^17^ proposed the idea of isolating the abdominal wound and the intestinal discharge from the fistulose surface. To carry out this isolation, different conventional devices have been used, such as a bottle nipple,^18^ a plastic roll of tape, wrapping it with gauze and placing Duoderm® in the base,^19^ and other commercial devices such as the PPM Fistula Adapter™.^20^, ^21^ The methods described so far isolate the intestinal content of the wound by creating a floating stroma over a NPWT device that assists in wound granulation. However, none of these devices consider the huge clinical variability in enteroatmospheric fistulas. Current 3D printing techniques facilitate device customization for clinical applications. Xu et al.^22^ described a 3D‐printed intraluminal stent that aims to restore intestinal continuity to reduce intestinal leakage. Although it seemed promising, their rates of intestinal leakage into the wound were unmanageable, particularly when enteral nutrition was added to the patient. The device we present does not intend to restore intestinal continuity, but to achieve fistula ostomization since no work has been found with successful results. Our device aims to isolate de wound from the intestinal content similar to previous studies,^17^, ^18^, ^19^, ^20^ with the additional advantages of personalized therapy in these patients. Although similar works^20^ have implemented devices for enteroatmospheric fistula treatment, the one presented in this article results considerably more advantageous, thanks to its potential personalization and its conical morphology, essential for the NPWT system.

For the manufacture of the device, PCL^23^, ^24^, ^25^ and polyamide^26^, ^27^ were used, one of the most widely used materials for 3D printing. PCL is approved by the Food and Drug Administration (FDA) of the United States.^24^ Previous studies have shown that this material stimulates the migration of muscle cells and the growth and proliferation of fibroblasts, chondrocytes, and mesenchymal stem cells, which leads us to think about its good behavior in healing.^23^, ^25^, ^28^ Polyamide is a medical‐grade biomaterial that has been utilized in surgical guides and instruments.^26^, ^27^ Some studies^29^, ^30^, ^31^ reported good properties of this biomaterial, such as attachment, proliferation, and migration of chondrocytes. However, these biomaterials are not flexible and they may cause discomfort to the patients and limit their movement.

Layton^18^ presented the use of a bottle nipple for isolating fistulas. This technique allows greater patient mobility due to the flexibility of the instruments. However, this device could not be applied in 75% of the cases presented due to the extensive surface occupied by the fistula and the large debit that was expelled. Our personalized device allows great mobility to the patients, avoiding muscle mass loss and enabling them to start early the motor rehabilitation usually needed in these cases.

Another of the problems encountered with these devices is that the PCL material does not adhere correctly to the NPWT system adhesives. To solve this, we lined the device with transparent polyurethane adhesive dressings (Opsite®, Smith, and Nephew), thus achieving an adherent surface on which to stick the NPWT system adhesives successfully. However, the use of polyamide devices did not show this drawback and allowed an optimal adhesion to the NPWT system.

The sterilization of the devices with hydrogen peroxide using Sterrad 100nx (Johnson & Johnson, USA) posed a challenge due to the device deformation caused by the temperatures and high pressures. However, using Sterrad 100S (Johnson & Johnson, USA), the devices maintained their shape. Our future research line could be focused on the improvement of the sterilization process of the devices. Another limitation of this methodology is the fact that a bioscanner with white light technology is not available in many hospitals, and therefore the externalization of this methodology is challenging.

## 4 MATERIALS AND METHODS

### 4.1 Actuation methodology design

Figure 3 shows the methodology established for the design and manufacture of the devices for enteroatmospheric fistulas treatment. The steps followed consist of the scanning of the wound and the point cloud generation, the 3D reconstruction of the wound and its measurement, the device design and manufacture, and finally its use on the patient. The process is described in detail in the following sections.

#### 4.1.1 Measurements taking and surface analysis

Two different approaches were compared: manual and digital measurement. The manual measurements were taken using a clean surgical ruler. For the digital measurements, a virtual reconstruction of the wound was achieved by scanning the enteroatmospheric fistula with the EinscanPro+ scanner (Shining 3D, China), which uses white light technology with a resolution between 0.1 and 0.3 mm.

Before taking images, the scanner must be calibrated, and due to the physiopathology of this medical condition, the patient must be placed in a supine position to perform the wound cleaning and scanning process with minimal movements. As a result of the scanning process, a point cloud was generated. Clouds with less than 400,000 points require a re‐scanning of the wound. To obtain a digital twin of the fistula, surface reconstruction was performed using a meshing procedure. High‐definition meshes were obtained using as many polygons as possible to achieve greater surface sharpness, but considering that the higher the number of polygons and triangles, the higher the computational cost and the processing time. At the end of this step, we obtained a digital twin of the wound that will be used afterward to design the final device. Once the surface is generated, it is exported to STL format.

#### 4.1.2 Device design

The scanner emits a beam of light that bounces off the surface and is picked up by the scanner's camera. Depending on the deformation of the beam of light picked up by the camera, the distance to the point on the surface where the light hit the surface is known, thus generating a point cloud and a virtual reconstruction of the wound and the fistula. Note that the design of the adapter is based on the image obtained from the surface of the wound. No internal images of the wound are obtained using the scanner. The 3D volume obtained represents the surface of the patient's body, including opening wounds such as those presented in patients with enteroatmospheric fistula.

The surface mesh obtained previously is used for the design of the customized adapter, considering the dimensions of the abdominal wound and the exposed intestinal surface of each patient, with the software FreeCAD 0.16® (FreeCAD© Juergen Riegel, Werner Mayer, Yorik van Havre). The dimensions were measured by the STL file, such as the height of the fistulous orifices, and these measurements gave us the dimension of the device. With the software Meshmixer, the fistulous surface was selected, measured, and extruded.

The design of the adapter (Supplementary video S1) is performed considering the extruded fistula surface as follows.

**FIGURE 4:**
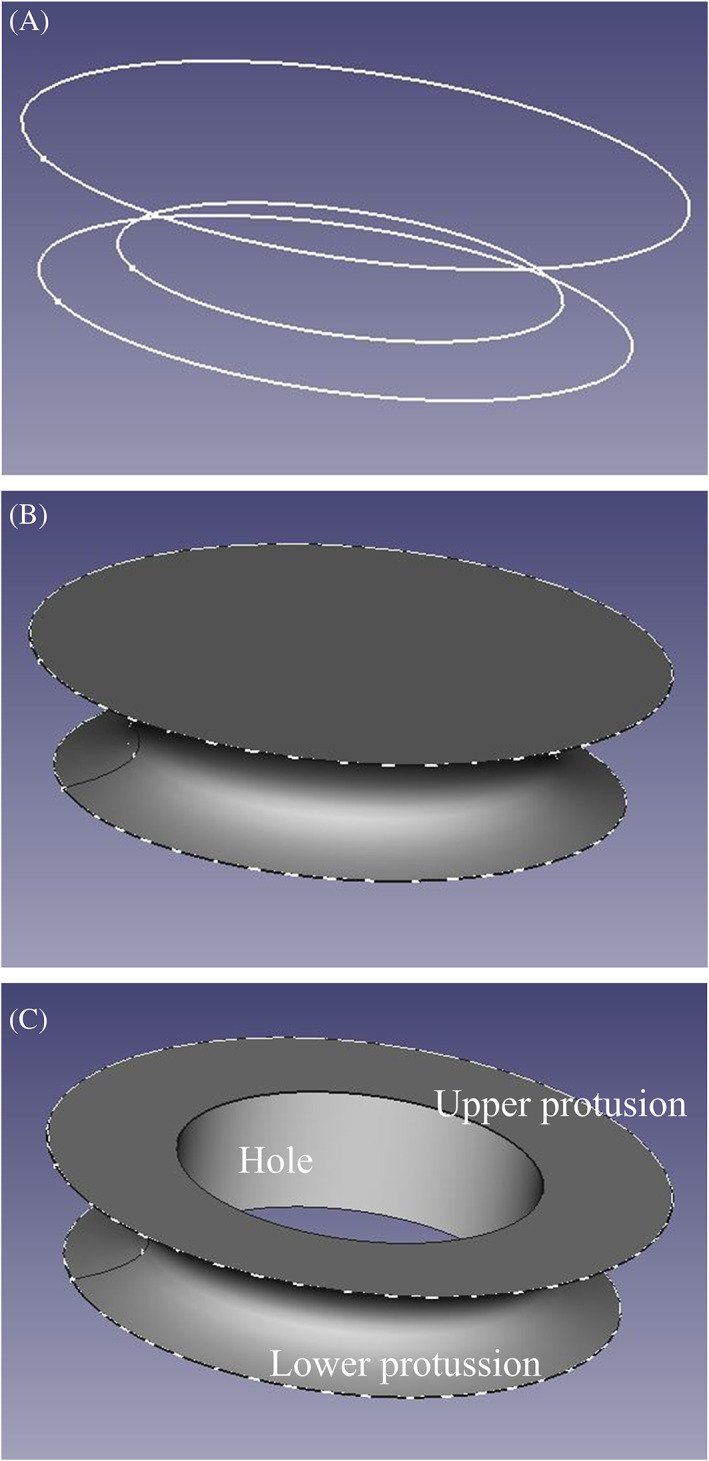
Device design. (a). Sketches in different planes. (b). Projection of three sketches. (c). Final design.

Our adapter (Figure 4) consisted of a model with a hollow interior and similar to a ring, to allow the exit of the extruded intestinal surface through the hole, which was unique for each case, having the same geometry and dimensions as the intestinal surface area of each fistula. Once the measurements were taken, a device was created with a base of the same geometry as the intestinal wound and between 1 and 2 cm in height, depending on the depth of each wound, that is, depending on the height of the adipose tissue and the height of the fistulous endings. To do this, three sketches were created in different planes (Figure 4a), one at 0, one at the maximum height of the device, and the other one between 15% and 25% of the height of the device. This distance depended on the height of the device; the lower the height, the greater the distance. The lower and upper planes had a radio of 0.5 cm and up to 2 cm more than the fistula surface, respectively. The lower surface is smaller to have a larger area of the wound in contact with the NPWT system and maximize the granulation of the wound. The upper one has the function of holding the polyurethane film that serves as support and helps to obtain the vacuum for the sponge of the NPWT system that is used with the device for the management of the wound. This surface varied with the distance between the fistula and the adipose wall, being smaller in cases where the fistula was closer to the adipose tissue. In some cases, the center of the upper sketch was even moved with respect to the center of the lower sketch. In this way, the intestinal discharge is to be conducted and the sponge could be placed between the device and the adipose tissue. The middle sketch is designed to ensure the fit with the fistulous surface and the NPWT system. Then, from the three 2D sketches, a projection was performed to generate the 3D prism (Figure 4b).

Finally, to create the hollow volume, the design was placed so that the extrusion of the fistula was in the center of the design in Meshmixer. A Boolean operation was performed, where the extrusion of the intestinal surface is subtracted from the created prism. The protrusion was defined as the difference between the outer and inner perimeter of the device in the upper and lower plane (Figure 4c). To check the assembly of the device with the fistula, the device design was placed over the wound scan. Given the inventive characteristics of this innovation, this device and its manufacture have been protected as a patent and utility model by the Spanish Patent and Trademark Office.

#### 4.1.3 Additive manufacturing

The designs were exported in STL files. “Regemat 3D designer®” software created the .gcode files from the STL files. Finally, the .gcode files were sent to a 3D printer (Regemat 3D®, Granada, Spain) to manufacture the devices. In our first attempts, polycaprolactone (PCL; 99 filament 750 g 1.75 mm, 3D4makers, Haarlem, The Netherlands) was used to print the device. The fill pattern was solid and a layer height of 0.35 mm was used. The printhead speed was 20 mm/s for the fill and 10 mm/s for the perimeter. The print flow was 1.8 mm/s at a temperature of 80°C. To improve our production process, we have included polyamide (PA2200, Moldkar, Spain), which improves de adherence to NPWT bandages, reducing the time for wound cures. Two devices of each design were manufactured and duly sterilized for each refill of the therapy performed on the patients.

#### 4.1.4 Post‐processing

After the manufacturing process, the obtained geometries present defects that must be manually eliminated in a post‐processing step to obtain a final device with the maximal adjustment. To do that, the printed devices were polished to remove any burrs left over from the manufacturing process.

#### 4.1.5 Sterilization of the manufactured devices

For their clinical application, it was necessary to sterilize or highly disinfect the devices. We tested the sterilization of the device with hydrogen peroxide at low temperatures using Sterrad 100S (Johnson & Johnson, USA) and Sterrad 100nx (Johnson & Johnson, USA). The first one was replaced at Virgen del Rocío Hospital, while the second one produced deformities. Therefore, instead of hydrogen peroxide, a high disinfection method was used by immersion for 15 minutes in Korsolex PAA from “BODE Chemie GmbH” (Hartmann Group, Hamburg‐Germany), a peracetic acid‐based disinfectant, indicated for surgical instruments (including thermoplastics). Then, the devices were washed with physiological saline to remove the residues of the disinfectant.

#### 4.1.6 Use of the device together with the NPWT

For the clinical application of the device along with the NPWT therapy, a sponge was molded to the shape of each patient's abdominal wound. We made an opening into the sponge of the NPWT system according to the size of the device, and in this gap, which was also located right above the intestinal surface, the device was placed. This way, we achieved a proper location of the intestinal surface inside de manufactured adapter, while the rest of the abdominal wound was covered by the sponge, and greater sealing of the wound was accomplished with an adhesive film. When the NPWT was connected at a pressure of 125 mmHg, a successful and concentrated granulation of the wound was achieved through the orifice of the device, below which we found the surface that needed exposition to the therapy, with minimal intervention on healthy tissue.

#### 4.1.7 Remodeling

When a cure was performed and some changes were appreciated in the wound as a result of the natural evolution of the disease, another scan was taken to check for any variation. When any considerable variation was perceived, the device was manufactured with the last measurements taken.

### 4.2 Outcomes

The main objective of this work is the design a methodology to personalize and manufacture a device for enteroatmospheric fistula treatment that adapts to the dimensions and morphology of each patient.

In addition to this, we aim to compare the time that manual and digital measurements require along the process, as well as the assessment of the efficacy of the personalized device in the treatment of enteroatmospheric fistula using NPWT via wound size, cures, pruritus, Visual Analogic Scale for pain (VAS) before and after therapy as well as the final result of each patient and survival.

### 4.3 Description of the cases

**FIGURE 5:**
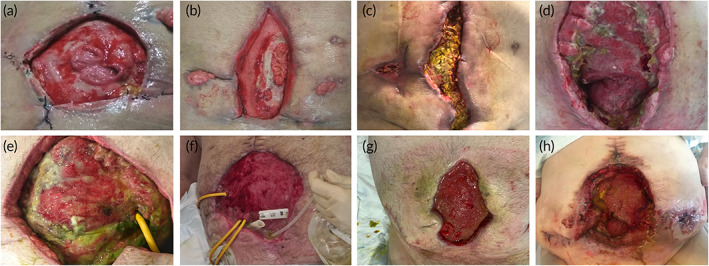
Fistula surface on the patient's abdominal wound. Patient at the beginning of the therapy (a) Patient 1; (b) Patient 2; (c) Patient 3; (d) Patient 4; (e) Patient 5; (f) Patient 6; (g) Patient 7; and (h) Patient 8.

From June 2017 to September 2022, all patients at our center over 18 years old diagnosed with enteroatmospheric fistula were prospectively preselected for the study. Exclusion criteria included: shapes or dimensions of the wound that did not allow customization of the device (e.g., a fistula very close to the edge of the wound), clinical conditions of the patient that prevented placement of the device (hemodynamic instability or need for urgent surgery), and not signing the informed consent. This study has been approved by the ethics committee (Research Ethics Committee of Seville). All experiments were performed in accordance with relevant named guidelines and regulations. Informed consent was obtained from all participants and/or their legal guardians, even to publish the information and images in an online open‐access publication. In this period, nine patients were shortlisted. Of these, eight were included in the study; one patient was excluded because she did not sign the informed consent. Table 2 presents the characteristics and evolutionary changes of the wounds. The parameters required to generate the device are: the initial dimensions of the abdominal wound where the fistula is located, the size and morphology of the fistulose surface, the disposition of the fistulose surface in relation to the rest of the wound, and the number of fistulose holes. Figure 5 shows a real image of a patient with a diagram of the fistulous surface dimensions.

### 4.4 Statistical analysis

Quantitative variables, depending on whether they followed or not a symmetrical distribution, were expressed as mean and standard deviation (M ± SD), or as median and interquartile range (P50 [P25–75]), respectively. The normal distribution of the sample was determined using the Shapiro–Wilks test if *n* < 50. For quantitative variables that followed a normal distribution, T‐Student's test was used, while for those quantitative variables that did not present a normal distribution, the Mann–Whitney *U* test was applied. The required significance was *p* < 0.05. For all statistical analyses, IBM® SPSS® Statistics 19 package was used.

## 5 CONCLUSIONS

In this work, we present a workflow for the design and manufacture of customized devices for enteroatmospheric fistula treatment coupled with NPWT therapy. The method proposed combines white light scanning, CAD design, and additive manufacturing, and provides the required devices in time with promising results.

## AUTHOR CONTRIBUTIONS

**Francisco José Calero Castro:** Conceptualization (equal); data curation (equal); formal analysis (equal); investigation (equal); methodology (equal); validation (equal); writing – original draft (equal). **Andrés Padillo Eguía:** Conceptualization (equal); methodology (equal); writing – review and editing (equal). **Virginia Durán Muñoz‐Cruzado:** Conceptualization (equal); data curation (equal); investigation (equal); supervision (equal); visualization (equal); writing – review and editing (equal). **Luis Tallón Aguilar:** Formal analysis (equal); investigation (equal). **José Tinoco González:** Formal analysis (equal); investigation (equal). **Imán Laga:** Writing – original draft (equal). **Fernando de la Portilla de Juan:** Funding acquisition (equal); resources (equal). **Felipe Pareja Ciuró:** Investigation (equal); supervision (equal); validation (equal); visualization (equal); writing – review and editing (equal). **Javier Padillo Ruiz:** Conceptualization (equal); funding acquisition (equal); resources (equal); supervision (equal); visualization (equal); writing – review and editing (equal).

## FUNDING INFORMATION

The contract of Francisco José Calero Castro was funded by Carlos III Health Institute (Health Research Fund) grant number PI19/01821.

## CONFLICT OF INTEREST STATEMENT

The authors declare no conflicts of interest.

### PEER REVIEW

The peer review history for this article is available at https://www.webofscience.com/api/gateway/wos/peer-review/10.1002/btm2.10583.

## Supporting information


**Data S1:** Supporting Information.

## Data Availability

Data available on request from the authors.
